# IL-27 induces LL-37/CRAMP expression from intestinal epithelial cells: implications for immunotherapy of *Clostridioides difficile* infection

**DOI:** 10.1080/19490976.2021.1968258

**Published:** 2021-08-25

**Authors:** Banglao Xu, Xianan Wu, Yi Gong, Ju Cao

**Affiliations:** a Department of Laboratory Medicine, Guangzhou First People’s Hospital, School of Medicine, South China University of Technology, Guangzhou, Guangdong, China; b Department of Laboratory Medicine, The First Affiliated Hospital of Chongqing Medical University, Chongqing, China; c Department of Blood Transfusion, The First Affiliated Hospital of Chongqing Medical University, Chongqing, China

**Keywords:** *Clostridioides difficile*, CRAMP, LL-37, colonic epithelial cells, IL-27, infection, immunity

## Abstract

*Clostridioides difficile* infection is currently the leading cause of nosocomial antibiotic-associated diarrhea and pseudomembranous colitis worldwide. Cathelicidins, a major group of natural antimicrobial peptides, have antimicrobial and immunomodulatory activities in *Clostridioides difficile* infection. Here, we have shown that cytokine IL-27 induced human cathelicidin antimicrobial peptide (LL-37) expression in primary human colonic epithelial cells. IL-27 receptor-deficient mice had impaired expression of cathelicidin-related antimicrobial peptide (CRAMP, mouse homolog for human LL-37) after *Clostridioides difficile* infection, and restoration of CRAMP improved *Clostridium difficile* clearance and reduced mortality in IL-27 receptor-deficient mice after *Clostridioides difficile* challenge. In clinical samples from 119 patients with *Clostridioides difficile* infection, elevated levels of IL-27 were positively correlated with LL-37 in the sera and stools. These findings suggest that IL-27 may be involved in host immunity against *Clostridioides difficile* infection via induction of LL-37/CRAMP. Therefore, IL-27-LL-37 axis may be a valuable pathway in the development of immune-based therapy.

## Introduction

*Clostridioides difficile* infection (CDI) is one of the most important hospital-acquired infectious diseases, which has been associated with a growing cause of human morbidity and mortality, especially after antibiotic use in healthy humans.^1,2^ It is important to develop new antimicrobial agents, especially immunotherapy targets, in the treatment of CDI.

Antimicrobial peptides (AMPs) are one of the first immunological barriers elicited during infection by various pathogens.^3^ Cathelicidin (CRAMP in mouse/rat, LL-37 in human), a major group of AMPs, also designated as host defense peptides, has emerged as a key component of innate immunity due to its direct antimicrobial activity against a broad spectrum of invading pathogens.^4^ The only human cathelicidin, LL-37, is released by proteolytic cleavage from an inactive 18-kDa precursor protein (hCAP-18), which is synthesized from epithelial cells, and also from a variety of other immune cells. The main mechanism of action of cathelicidins is the formation of an amphipathic alpha-helical structure that can insert into bacterial membranes and cause bacterial cell death, but they can also act on intracellular targets, such as nucleic acid and protein biosynthesis.^3,4^ Low baseline levels of LL-37 have been shown to be independently associated with an increased risk of death attributable to infection in patients undergoing hemodialysis.^5^ Cathelicidins have been shown to inhibit *C. difficle* growth,^6,7^ and they could modulate *C. difficle*-associated colitis and toxin A-mediated enteritis in mice.^8^ In addition, LL-37 and human Beta defensin 3 (hBD3) could act in synergy with tigecycline, moxifloxacin, piperacillin-tazobactam, and meropenem against CDI.^9^

Intestinal epithelial cells protect the host against pathogenic bacteria not only by forming a physical barrier but also by secreting many mediators such as cytokines, chemokines, and antimicrobial peptides.^10,11^ One important mechanism for the prevention of *C. difficle* invasion of the epithelium is the production and secretion of CRAMP/LL-37. However, it is expressed only at low and constitutive levels in the intestinal epithelium, and its expression is variously regulated during infection condition.^12,13^ Therefore, elucidating the molecular mechanisms regulating the expression of CRAMP/LL-37 from intestinal epithelial cells may have important implications in the treatment of CDI.

Cytokine-elicited immunity contributes to innate and adaptive immunities against microbial infections.^14^ Interleukin (IL)-27 is a new member of the IL-6/IL-12 family cytokines that are primarily produced by antigen-presenting cells (APC) after stimulation of APC by Toll-like receptor (TLR) ligands or infectious agents.^15^ IL-27 regulates infection and immunity upon binding to a heterodimeric receptor complex consisted of T-cell cytokine receptor (TCCR)/WSX-1 and glycoprotein 130 (gp130), the former confers ligand specificity and the latter is shared by other IL-6 family cytokines.^15^ We have recently shown that IL-27 protein levels were increased in the sera and cecal tissues after murine CDI, and IL-27 contributed to host defense to murine CDI.^16^ However, the mechanism by which IL-27 protected against CDI require further studies, especially the role of cathelicidin in IL-27-mediated protection against CDI remains unknown. Given the importance of intestinal epithelial cells in host defense against CDI, we hypothesized that IL-27 may exert direct effects on intestinal epithelial cells to initiate antimicrobial defense during CDI. We investigated here the contribution of IL-27 to stimulate the expression of LL-37 and its murine homologue, CRAMP, utilizing primary human colonic epithelial cells and murine CDI. Our results demonstrated that IL-27 stimulated synthesis of CRAMP/LL-37 in intestinal epithelial cells, suggesting that IL-27-LL-37 axis may be a valuable immunotherapy target for human CDI.

## Results

### IL-27 induced LL-37 expression in primary human colonic epithelial cells

**Figure 1.**
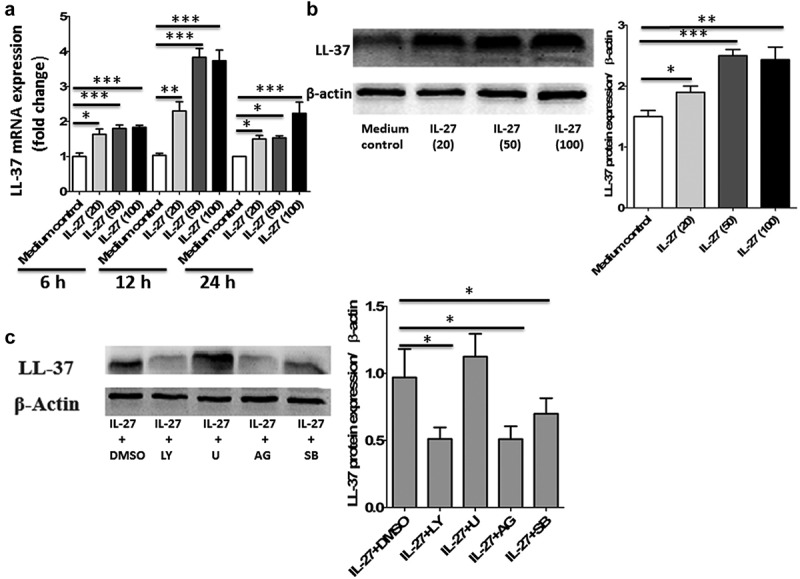
IL-27 induced LL-37 expression in primary human colonic epithelial cells. (a) Kinetic gene expression of LL-37 in primary human colonic epithelial cells after stimulation with different doses of recombinant human IL-27 protein (20, 50 and 100 μg/ml) for different times (6, 12 and 24 hours). (b) Representative Western blot analysis of LL-37 protein expression in cell lysates of primary human colonic epithelial cells at 24 hour after stimulation with different doses of recombinant human IL-27 protein (20, 50 and 100 μg/ml). Total proteins were extracted from primary human colonic epithelial cells (1 × 10^6^ cells), and an equal amount of protein (10 μg) was subjected to SDS-PAGE (10%) before blotting onto a PVDF membrane. β-Actin was used as a control to ensure an equal amount of loaded protein. (c) Effects of signaling molecule inhibitors on LL-37 protein expression. Primary human colonic epithelial cells were pretreated with AG490 (5 μM; AG), LY294002 (10 μM; LY), SB203580 (20 μM; LY), or U0126 (10 μM; U) for 1 hour, followed by incubation for 24 hour with or without IL-27 (50 ng/ml). LL-37 protein expression in cell lysates was analyzed by Western blot. Densitometry quantification of blots was shown as in histograms on the right. LL-37 expression was normalized to β-actin for each sample, and expression was graphed as fold change above cells at 0 min. Results are expressed as mean ± SD of 5 independent experiments. *P < .05, **P < .01,***P < .001 when compared between groups denoted by horizontal lines

In the gut, LL-37 is constitutively expressed in the colon by the colonic epithelium.^13^ To examine whether IL-27 is able to induce LL-37 expression, primary human colonic epithelial cells were stimulated with varying doses of IL-27 at different time points, and the levels of LL-37 gene and protein expression were analyzed. As shown in Figure 1a, IL-27 induced LL-37 gene expression by quantitative polymerase chain reaction (PCR) in human colonic epithelial cells time-dependent (0–24 h) and dose-dependent (0–100 ng/ml). Furthermore, the protein expression of LL-37 by Western blot in cell lysates was significantly increased after stimulation with IL-27 when compared to medium control (Figure 1b).

To investigate IL-27-mediated signaling events leading to the induction of LL-37, primary human colonic epithelial cells were pretreated with different selective signaling molecule inhibitors. The cytotoxicities of signaling molecule inhibitors on human colonic epithelial cells have been determined by methylthiazolyldiphenyl-tetrazolium bromide (MTT) assay (data not shown), and the specificities of these inhibitors on their signaling pathways have been investigated by using human epithelial cells in our previous studies.^17^ Following our studies and toxicity threshold values from the MTT assay, we selected the optimal concentrations of Janus kinase (JAK) inhibitor AG490 (5 μM), phosphatidylinositol 3-OH kinase (PI3K) inhibitor LY294002 (10 μM), extracellular signal-regulated kinase (ERK) inhibitor U0126 (10 μM), and p38 mitogen-activated protein kinase (MAPK) inhibitor SB203580 (20 μM) with significant inhibitory effects but without any cell toxicity. As shown in Figure 1c, AG490 and LY294002 could potently suppress IL-27-induced LL-37 production, whereas SB203580 could partially but significantly suppress LL-37 production in primary human colonic epithelial cells stimulated by IL-27. However, U0126 did not exert any significant effect on LL-37 production induced by IL-27. Collectively, these results suggest that the augmenting production of LL-37 in human colonic epithelial cells stimulated by IL-27 may be regulated by the activation of JAK and PI3K signaling pathways primarily and at least in part via the activation of p38MAPK signaling pathway, while ERK signaling pathway may have no role in IL-27-induced LL-37 production.

### IL-27 signaling was required for CRAMP up-regulation during murine CDI

**Figure 2.**
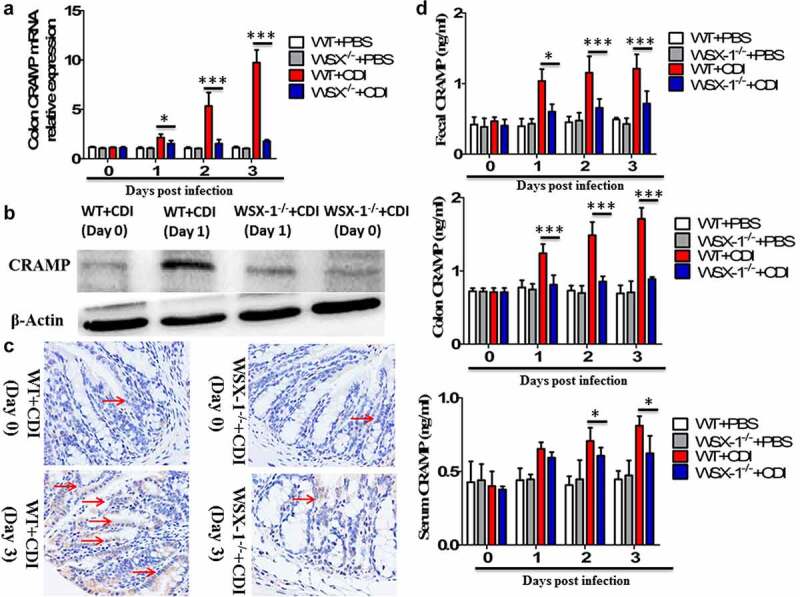
Lack of IL-27 signaling impaired CRAMP expression in murine CDI. Wild-type (WT) and IL-27 receptor-deficient (WSX-1^−/−^) mice were infected with 10^4^ colony-forming units of *C. difficile* VPI 10463 after antibiotic pretreatment. (a) At the indicated time after *C. difficile* infection, mice were sacrificed and ceca were harvested. Then total cellular RNA was extracted, and mRNA expression of CRAMP in colon tissues from mice (12 mice per group) was analyzed by quantitative PCR after *C. difficile* challenge. Details are described in the online supplementary methods. (b) Representative Western blot analysis of CRAMP protein expression in colon tissues from mice (12 mice per group) at day 0 or 1 after *C. difficile* challenge. (c) Immunohistochemistry by standard methods with anti-CRAMP antibody on fixed colon tissues of mice (12 mice per group) at day 0 or 3 after *C. difficile* challenge. Details are described in the online supplementary methods. Red arrows point to areas of CRAMP staining (brown). (d) CRAMP protein levels quantified by ELISA in the stools, colon tissues and sera of mice after *C. difficile* challenge. Details are described in the online supplementary methods. Results are expressed as mean ± SD of 3 independent experiments. *P < .05, ***P < .001 when compared between groups denoted by horizontal lines

**Figure 3.**
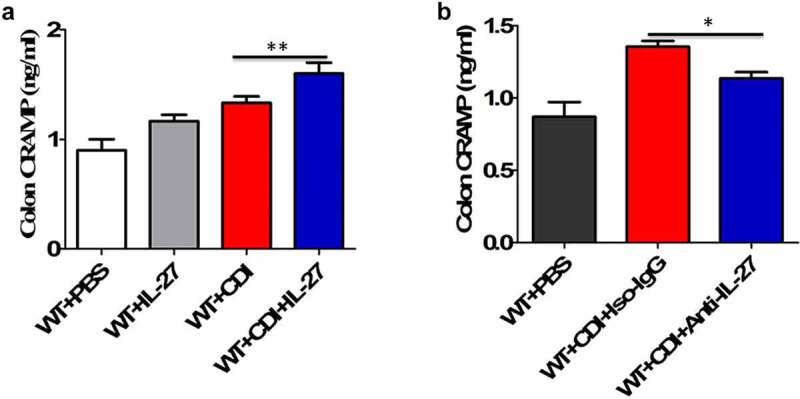
The effects of IL-27 administration or IL-27 blockade on CRAMP production. (a) CRAMP protein levels at day 1 in the colon tissues from mice (12 mice per group) treated with or without recombinant mouse IL-27 protein (0.5 μg/mice) immediately after *C. difficile* challenge. Phosphate buffer saline (PBS) was used as vehicle control. (b) CRAMP protein levels at day 1 in the colon tissues from mice (12 mice per group) treated with or without goat anti-IL-27 polyclonal IgG antibodies (100 μg/mice) immediately after *C. difficile* challenge. Polyclonal goat IgG was used as isotype control. Results are expressed as mean ± SD of 3 independent experiments. *P < .05, **P < .01 when compared between groups denoted by horizontal lines

We next used a well-established murine model of CDI, produced by infection of antibiotic-treated WT and IL-27-receptor knockout (WSX-1^−/−^) mice with *C. difficile* VPI 10463. After infection, WSX-1^−/−^ mice showed significantly reduced gene (Figure 2a) and protein (Figure 2b) expression of CRAMP in the colonic tissues when compared to wild-type (WT) mice. Furthermore, anti-CRAMP antibodies were used to stain colon tissue sections from WT and WSX-1^−/−^ mice for immunohistochemistry (IHC) analysis. Although there was detectable CRAMP staining in WSX-1^−/−^ mice after CDI, we observed increased levels of CRAMP staining in the colonic epithelium of WT mice when compared to WSX-1^−/−^ mice after CDI (Figure 2c). In addition, ELISA results confirmed that significantly lower protein levels of CRAMP were observed in the stools and colon tissue homogenates as well as serum samples of WSX-1^−/−^ mice when compared to those of WT mice (Figure 2d). Finally, administration with recombinant IL-27 protein in *C. difficile*-infected WT mice significantly enhanced CRAMP production in the colonic tissues compared to control mice (Figure 3a), while treatment with anti-IL-27 blocking antibodies significantly decreased CRAMP production in *C. difficile*-infected WT mice compared to Isotype IgG (Figure 3b).

### The CRAMP peptide reduced the susceptibility to CDI in IL-27-receptor knockout mice

**Figure 4.**
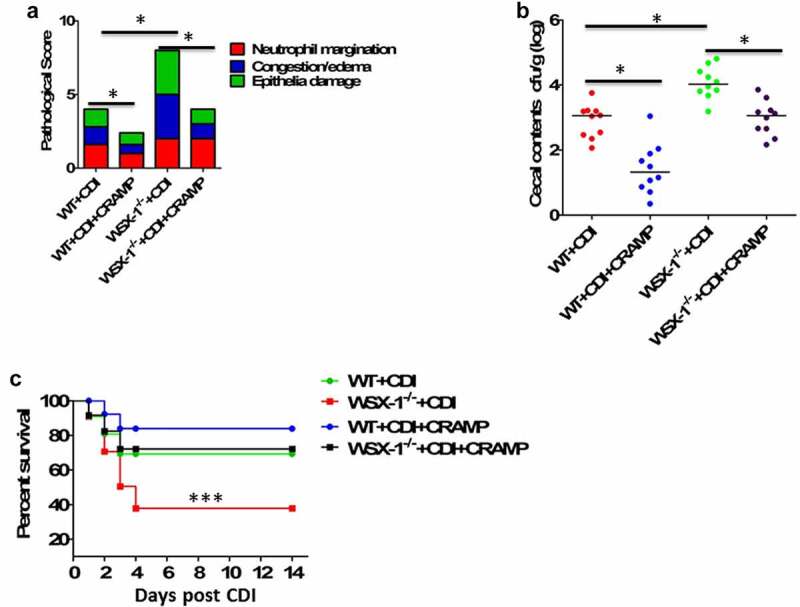
Exogenous CRAMP reduced *C.difficile* infection-associated mortality and morbidity in IL-27 receptor-deficient mice. WSX-1^−/-^ mice were treated with or without CRAMP peptide 2 hours after infection with of *C. difficile*. (a) Tissue pathology scores of cecal tissues from WSX-1^−/-^ mice (10 mice per group) treated with or without CRAMP peptide. Data were expressed as mean ± SD. *P < .05 when compared between groups denoted by horizontal lines. (b) *C. difficile* bacterial burden in cecal contents from WSX-1^−/-^ mice (10 mice per group) on day 3 post *C. difficile* infection. Horizontal bars represent median values, and dots represent individual mouse. *P < .05 when compared between groups denoted by horizontal lines ((Mann–Whitney U test). (c) Survival was monitored until 2 weeks postinfection (12 mice per group). Comparison between groups was done by Kaplan-Meier analysis followed by log-rank tests. ***P < .001 when compared with WSX-1^−/-^ mice treated with CRAMP peptide

Our previous study has demonstrated that IL-27 receptor-deficient (WSX-1^−/−^) mice were defective in the clearance of *C. difficile*, and they displayed increased disease severity and higher mortality compared to WT mice.^16^ Having observed that stimulatory effects of IL-27 on production of LL-37, along with decreased levels of CRAMP (mouse homolog for human LL-37) in WSX-1^−/−^ mice during CDI, we next examined whether injection of exogenous CRAMP peptide into ileal loops may reduce mortality and morbidity in WSX-1^−/−^ mice after CDI. The concentration of CRAMP peptide in ileal loop treatment is equivalent to 10 mg/kg, previously shown to successfully reduce tumor necrosis factor (TNF)-α expression during *C.difficile* infection and toxin A-mediated inflammation in mice.^8^ Of note, providing CRAMP peptides could significantly decrease CDI-associated tissue pathology in WSX-1^−/−^ mice (Figure 4a). CRAMP treatment in *C. difficile*-infected WSX-1^−/−^ mice also significantly lowered the density of *C. difficile* in the cecum (Figure 4b). Importantly, CRAMP supplementation was capable of reducing CDI-associated mortality in WSX-1^−/−^ mice when compared with control mice (Figure 4c).

### IL-27 and LL-37 were clinically relevant in C. difficile patients

Next, we wondered whether the IL-27-LL-37 axis was relevant in human *C. difficile* infection. A total of 119 CDI-positive and 63 CDI-negative patients were enrolled in this study. Baseline patient characteristics were described in supplementary Table 1. Severe CDI was defined with a white blood cell (WBC) count >15,000 per microliter within 48 h of CDI diagnosis as previously described.^18^ Significant increases in serum and fecal IL-27 protein concentrations were observed in CDI-positive patients when compared to CDI-negative patients (Supplementary Figure S1A). Interestingly, when CDI-positive patients were categorized based on disease severity, those with severe CDI had significantly lower serum and fecal IL-27 levels compared to those with non-severe CDI (Supplementary Figure S1B). In addition, IL-27 levels correlated positively with LL-37 in the sera (Supplementary Table S2) and stools (Supplementary Table S3) of CDI patients. However, there was no apparent correlation between IL-27 and IL-1β, TNF-α, IL-6, or IL-8 in the sera and stools.

## Discussion

In this report, we demonstrated for the first time that IL-27 induced LL-37 expression in primary human colonic epithelial cells *in vitro*, and lack of IL-27 signaling impaired CRAMP production in an *in vivo* CDI mouse model. More interestingly, up-regulated levels of IL-27 were positively correlated with LL-37 in the sera and stools of CDI patients. Our results therefore provided a novel functional linkage between IL-27 and LL-37/CRAMP, suggesting that IL-27-LL-37 axis might be a therapeutically important pathway in the treatment with human CDI.

LL-37/CRAMP, a cationic antimicrobial peptide, plays a key role in initial immune response to bacterial infections in the intestinal tract.^3^ LL-37/CRAMP is constitutively and restrictedly expressed by colon surface epithelium in the gut, and epithelial LL-37/CRAMP is of functional importance as a component of early innate antimicrobial defense in the colon.^12,19^ A decrease in epithelial LL-37/CRAMP could result in increased bacterial infections in the colon.^18^ Here, we provided evidence that IL-27 is a novel inducer of LL-37 in primary human colonic epithelial cells, which may be regulated by the activation of JAK and PI3K signaling pathways primarily and at least in part via the activation of p38MAPK signaling pathway. Intracellular signal transduction is a highly interactive network composed of various types of protein kinases and messenger cascades.^19^ The cross-talk between discrete intracellular signaling pathways may precisely regulate IL-27-induced LL-37 production in human colonic epithelial cells. However, further experiments are required to investigate the regulatory mechanisms of different signaling pathways for the induction of LL-37 in human colonic epithelial cells upon IL-27 stimulation.

Murine CRAMP and human LL-37 manifest the same restricted distribution to surface epithelial cells in the colon.^3,20^ In a murine model of CDI, we also found that loss of IL-27 signaling led to a significant decrease of CRAMP expression in the colonic epithelium. Furthermore, local and systemic release of CRAMP was reduced in IL-27 receptor-deficient (WSX-1^−/−^) mice after CDI. In clinical samples of CDI patients, increased concentrations of IL-27 were positively associated with LL-37. Since LL-37/CRAMP has been demonstrated to possess both anti-bacterial and anti-inflammatory effects in CDI,^6,8,9^ uncovering interactions between IL-27 and LL-37/CRAMP might be valuable in understanding and treating CDI.

Consistent with our previous report,^16^ we found that IL-27 production was elevated in the sera and stools in a large cohort study of CDI patients. We hypothesize that CDI may up-regulate IL-27 production directly by bacterial components and indirectly by host factors, such as other cytokines.^14,15^ Interestingly, when patients were categorized based on disease severity, those with severe CDI had significantly lower serum and fecal IL-27 levels compared to those with non-severe CDI, suggesting that severe CDI might cause reduced IL-27 production from APC or other immune cells. In fact, IL-27-mediated intestinal immunity has been shown to play a protective role in the pathogenesis of CDI.^16^ In this study, we firstly found that IL-27–mediated host defense against CDI could be explained at least in part by the induction of LL-37/CRAMP. However, LL-37/CRAMP is also expressed in neutophils, monocytes and lymphocytes.^3^^,^^21^ Future experimentation is needed to understand whether IL-27 is able to induce LL-37/CRAMP expression in these leukocytes. Since LL-37/CRAMP expression by colonic epithelial cells could be induced by various molecules including the short chain fatty acid butyrate,^22^ it would be interesting to compare the potency of IL-27 and butyrate at inducing LL-37/CRAMP in the future studies.

In conclusion, this study has revealed that IL-27 induced the expression of an important antimicrobial peptide, LL-37/CRAMP, in colonic epithelial cells, which was involved in IL-27-mediated protection against CDI. Activation of the IL-27-LL-37/CRAMP axis may contribute to host immunity to CDI and have important therapeutic implications.

## Material and methods

Details of all of the Materials and Methods are provided in the online supplement.

### Bacterial culture

*C. difficile* strain VPI 10463 (ATCC 43255) was cultured and maintained at 37°C in beef heart infusion supplement (BHIS) media (BD Biosciences, Franklin Lakes, NJ) in an anaerobic environment (BBL GasPak Plus system; BD Biosciences). *C. difficile* were then subcultured under the same conditions for 5 hours before infection.

### Reagents

Recombinant human IL-27 (Catalog number: 2526-IL-010/CF) or mouse (Catalog number: 2799-ML-010/CF) IL-27 protein was purchased from R&D Systems (Minneapolis, MN). Mouse CRAMP (mouse homolog for human LL-37, Catalog number: SP-CRPS-5) was bought from Innovagen AB. The ERK inhibitor U0126 (Catalog number: 19–147), p38 MAPK inhibitor SB203580 (Catalog number: 559389), PI3K inhibitor LY294002 (Catalog number: 19–142), and JAK inhibitor AG490 (Catalog number: 658408) were purchased from Calbiochem (San Diego, CA). In all studies, the concentration of DMSO was 0.1% (vol/vol).

### Culture of human colonic epithelial cells

Primary human colonic epithelial cells (Catalog number: 2950) from ScienCell Research Laboratories (https://www.sciencellonline.com/, Carlsbad, CA) were isolated from human colonic tissue. Primary human colonic epithelial cells are characterized by immunofluorescence with antibodies specific to CK18 and CK19. Primary human colonic epithelial cells are guaranteed to further culture for 10 population doublings under the conditions provided by ScienCell Research Laboratories. Primary human colonic epithelial cells were cultured in Colonic Epithelial Cell Medium (Catalog number: 2951, ScienCell Research Laboratories) before stimulation.

### CDI mouse model

C57BL/6 mice aged 6–8 weeks were obtained from Chongqing Medical University (Chongqing, CQ). IL-27 R–deficient (WSX-1^−/−^) mice raised on C57BL/6 background were purchased from the Jackson Laboratory (Bar Harbor, ME USA). A characteristic murine CDI model was established as described in our previous study.^16^ Briefly, C57BL/6 mice received kanamycin (40 mg/kg), gentamicin (5 mg/kg), colistin (4 mg/kg), metronidazole (20 mg/kg), and vancomycin (5 mg/kg) in their drinking water for 3 days followed by 2 days of fresh water and a subsequent single intraperitoneal injection of clindamycin (10 mg/kg) 1 day prior to infection with *C. difficile* VPI 10463. All animal experiments were done in accordance with the Institutional Animal Care and Use Committee’s guidelines at the Chongqing Medical University (https://sydwzx.cqmu.edu.cn/).

### Patient population and clinical samples

We approached all hospitalized patients who had diarrhea (either on admission or during their hospitalization) between 2015 and 2019, and obtained written informed consent. Positivity of CDI was confirmed by Xpert *C. difficile* PCR (Cepheid, CA, USA) on stool. This study evaluated patients with newly diagnosed CDI, and patients with a history of CDI within 90 days were excluded. Demographics (age, gender, and race) and clinical characteristics, such as white blood cell counts, Charlson comorbidity index, medications, were extracted from the electronic health record database. CDI-negative samples were derived from patients suspected of various other intestinal diseases but confirmed negative for tissue pathology upon colonoscopy examination. These CDI-negative patients were actually diagnosed as transient diarrhea related to laxatives or tube feeds. We retrieved blood and stool specimens submitted to the microbiology laboratory for testing within 48 hours of submission. Blood and stool specimens were aliquoted and stored at −80°C upon receipt. This study was conducted in the first affiliated hospital of Chongqing Medical University, and approved by the Institutional Review Board of the first affiliated hospital of Chongqing Medical University (Approval number: 2017–0031).

### Statistical analysis

All experimental data were analyzed using SPSS (https://www.ibm.com/analytics/spss-statistics-software) and presented as mean ± SD using GraphPad Prism 7.0 unless otherwise stated. An independent-sample t test was used for comparison between two groups. One-way ANOVA followed by Bonferroni’s post hoc test was used for comparison among more than three groups. For mouse study, survival curves were compared using Kaplan–Meier analysis followed by a log rank test.^16^ For human patient study, data were expressed as box-and-whisker diagrams unless indicated otherwise. Differences between CDI-positive and CDI-negative patients were analyzed by Mann–Whitney *U* test.^16^ Correlation analysis was analyzed using the Spearman’s method. *P* value less than 0.05 was considered statistically significant.^23^

## Supplementary Material

Supplemental Material

## Data Availability

All data generated or analyzed during this study are included in this published article and its supplementary information files.
