# Supplementary material for: IL-27 induces LL-37/CRAMP expression from intestinal epithelial cells: implications for immunotherapy of Clostridioides difficile infection
Source: Gut Microbes. 2021 Aug 25;13(1):1968258. doi: 10.1080/19490976.2021.1968258 (PMC8405154; doi:10.1080/19490976.2021.1968258)
Supplement: Supplemental Material [file KGMI_A_1968258_SM1813.zip › Supplementary information/Supplementary Figure captions.docx]

**Supplementary Figure 1.** IL-27 production was elevated in the patients with *C.difficile* infection. (A) Sera and fecal IL-27 concentrations quantified by ELISA in the *C. difficile*-positive and *C. difficile*-negative patients. (B) Sera and fecal IL-27 levels for patients with non-severe and severe *C.difficile* infection. Horizontal bars represent median values, and dots represent individual participants. **P*<0.05, ***P*<0.01, ****P*<0.001 when compared between groups denoted by horizontal lines.

**Supplementary Table 1.** Demographic and clinical characteristics of the study patients.

**Supplementary Table 2.** Correlation between serum IL-27 and cytokine levels at the time of CDI diagnosis.

**Supplementary Table 3.** Correlation between fecal IL-27 and cytokine levels at the time of CDI diagnosis.
