# Supplementary material for: IL-27 induces LL-37/CRAMP expression from intestinal epithelial cells: implications for immunotherapy of Clostridioides difficile infection
Source: Gut Microbes. 2021 Aug 25;13(1):1968258. doi: 10.1080/19490976.2021.1968258 (PMC8405154; doi:10.1080/19490976.2021.1968258)
Supplement: Supplemental Material [file KGMI_A_1968258_SM1813.zip › Supplementary information/Supplementary Materials and Methods.docx]

**Supplementary Materials and Methods**

***Ileal CRAMP treatment in vivo***

Mice were firstly anaesthetised with isoflurane, and ileal loops (2–3 cm) were formed by tying up with surgical sutures. Mouse CRAMP (mouse homolog for human LL-37, Catalogue number: SP-CRPS-5) in phosphate buffer saline (PBS, total volume 200 μl per loop) was injected into ileal loops. The concentration of CRAMP in ileal loop treatment is equivalent to 10 mg/kg. The abdomen was then sealed by surgical sutures and wound clips, and the mice were returned to consciousness.

***Antibody-Mediated Neutralizations in vivo***

IL-27 neutralization was performed by intraperitoneal administration of 100 µg of goat anti-IL-27 polyclonal IgG antibodies (Catalogue number: AF1834, R&D systems) immediately after *C. difficile* infection as described previously.^1^ Polyclonal goat IgG (Catalogue number: AB-108-C, R&D systems) was used as isotype control.

***Endotoxin-free solutions***

Cell culture medium was purchased from Gibco Invitrogen Corp (Carlsbad, CA), free of detectable lipopolysaccharide (<0.1EU/ml). All other solutions were prepared using pyrogen-free water and sterile polypropylene plastic ware. No solution contained detectable LPS, as determined by the Limulus amoebocyte lyase assay (sensitivity limit 12 pg/ml; Biowhittaker Inc, Walkersville, MD).

***Quantitative reverse transcriptase* *polymerase chain reaction (PCR) for LL37/CRAMP expression***

Ribonucleic acid (RNA) was isolated according to the protocol supplied with the TRIzol reagent. Isolated mRNA (1 μg) was reverse transcribed into cDNA using the PrimeScript RT reagent kit with gDNA Eraser (Perfect Real Time) (TaKaRa). Quantitative real-time PCR (qPCR) was performed using SYBR premix Ex Taq II (TaKaRa) and the following primers: LL-37 (forward): 5’**-**ACCCAGCAGGGCAAATCTC-3’, reverse: 5’**-**GAAGGACGGGCTGGTGAAG-3’; human glyceraldehyde-3-phosphate dehydrogenase (GAPDH, forward): 5’**-**TCCTTGGAGGCCATGTGGGCCAT-3’, reverse: 5’**-**TGATGACATCAAGAAGGTGGTGAAG-3’; CRAMP (forward): 5’**-**GCTGTGGCGGTCACTATCAC-3’ and reverse: 5’**-**TGTCTAGGGACTGCTGGTTGA-3’; murine GAPDH (forward): 5’**-**AGGTCGGTGTGAACGGATTTG-3’, reverse: 5’**-**TGTAGACCATGTAGTTGAGGTCA-3’. Amplification efficiencies were validated and normalized against GAPDH. Quantification was determined using both a standard curve and comparative ΔΔCt methods. Each data point was examined for integrity by analysis of the amplification plot. The mRNA-normalized data were expressed as relative gene mRNA in treated compared to untreated experimental groups. ^2^

***Histopathology***

At the indicated time after *C. difficile* infection, mice were sacrificed and colons were harvested as previously described. ^3^ Colons were then fixed (1% ZnSO4, 10% formalin) and embedded in paraffin. Sections (5 μm) were treated with 0.3% H2O2 in PBS for 10 min at room temperature. For CRAMP staining, sections were incubated with goat serum (2%) and BSA (2%) for 1 h at room temperature, followed by incubation overnight at 4°C with 2 μg/ml rabbit anti-CRAMP IgG (Catalogue number: ab180760, Abcam) or 2 μg/ml preimmune IgG. HRP-labeled goat anti-rabbit IgG was used as secondary antibody. 3,3^,^-Diaminobenzidine (Sigma-Aldrich) was used for visualization. Sections were counterstained with hematoxylin.

***Western blot analysis***

Cells or tissues were washed with ice-cold PBS and lysed in 0.2 ml lysis buffer (20 mM Tris–HCl, pH 8.0, 120 mM NaCl, 1% Triton X-100, 10 mM EDTA, 1 mM EGTA, 0.05% 2-mercaptoethanol, 1 x protease inhibitors). Cell debris was removed by centrifugation at 14 000 g for 15 min, and the supernatant was boiled in Laemmli sample buffer (Bio-Rad Laboratory, Hercules, CA) for 5 min. An equal amount of protein (10 μg) was subjected to sodium dodecyl sulfate (SDS)-10% polyacrylamide gel electrophoresis (PAGE) before blotting onto a polyvinylidene fluoride (PVDF) membrane (Amersham and Pharmacia Biotech). The membrane was blocked with 5% skimmed milk in Trisbuffered saline with 0.05% Tween-20 for 1 hr at room temperature, and probed with anti-LL-37 (Catalogue number: ab207758, Abcam), or anti-CRAMP (Catalogue number: ab93357, Abcam) antibodies at 4° overnight. After washing, the membrane was incubated with corresponding secondary sheep anti-rabbit or sheep anti-mouse antibodies coupled to horseradish peroxidase (Amersham Pharmacia Biotech) for 1 hr at room temperature. Antibody–antigen complexes were then detected using an ECL chemiluminescent detection system. To assess protein expression, band intensity was quantified using IMAGEJ software (NIH, Bethesda, MD).

***Enzyme linked immunosorbent assay (ELISA)***

The concentrations of murine CRAMP (Catalogue number: CSB-E15061m) and human LL-37 (Catalogue number: CSB-E14948h) were quantified by ELISA (CUSABIO).^4^The levels of human IL-1β (Catalogue number: KAC1211), TNF-α (Catalogue number: KHC3013), IL-6 (Catalogue number: KAC1261), IL-8 (Catalogue number: KHC0081), and IL-27 (Catalogue number:  EH271RBX10 ) were measured by ELISA (Ebioscience), according to the manufacturer’s instructions.^5^

***Pathology score assessment***

At the indicated time after *C. difficile* infection, mice were sacrificed and cecal tissues were harvested. Tissue was fixed for 24 hours in Bouin’s solution, washed, and then stored in 70% ethanol prior to processing for hematoxylin-eosin (H&E) staining. The tissue was sectioned onto slides and scored by two independent, blinded observers. Each sample was given a score of 0–3 for each of the following parameters: epithelial disruption, submucosal edema, inflammatory infiltrate, mucosal thickening and luminal exudates as described previously. ^5, 6^

***C. difficile quantification***

Cecal contents were suspended in deoxygenated phosphate buffered saline (PBS), and 10-fold dilutions of the suspension were plated anaerobically on beef BHIS plates containing taurocholate, d-cycloserine, and cefoxitin for specific selection of *C. difficile*. Plates were placed in a 37°C incubator within the anaerobic chamber overnight. Colony forming units (CFUs) of *C. difficile* were counted and normalized to stool weight.

**References**

1. Cao J, Xu F, Lin S, Song Z, Zhang L, Luo P, Xu H, Li D, Zheng K, Ren G, Yin Y. IL-27 controls sepsis-induced impairment of lung antibacterial host defence. Thorax. 2014; 69(10): 926-937.

2. Dong S, Zhang X, He Y, Xu F, Li D, Xu W, Wang H, Yin Y, Cao J. Synergy of IL-27 and TNF-α in regulating CXCL10 expression in lung fibroblasts. Am J Respir Cell Mol Biol. 2013; 48(4): 518-530.

3. Buonomo EL, Cowardin CA, Wilson MG, Saleh MM, Pramoonjago P, Petri WA Jr. Microbiota-Regulated IL-25 Increases Eosinophil Number to Provide Protection during Clostridium difficile Infection. Cell Rep. 2016; 16(2): 432-443.

4. Bei Y, Pan LL, Zhou Q, Zhao C, Xie Y, Wu C, Meng X, Gu H, Xu J, Zhou L, Sluijter JPG, Das S, Agerberth B, Sun J, Xiao J. Cathelicidin-related antimicrobial peptide protects against myocardial ischemia/reperfusion injury. BMC Med. 2019;17(1):42.

5. Wang L, Cao J, Li C, Zhang L. IL-27/IL-27 Receptor Signaling Provides Protection in Clostridium difficile-Induced Colitis. J Infect Dis. 2018; 217(2): 198-207.

6. Hing TC, Ho S, Shih DQ, Ichikawa R, Cheng M, Chen J, Chen X, Law I, Najarian R, Kelly CP, et al. The antimicrobial peptide cathelicidin modulates Clostridium difficile-associated colitis and toxin A-mediated enteritis in mice. Gut. 2013; 62(9): 1295-1305.
