# Supplementary material for: IL-27 induces LL-37/CRAMP expression from intestinal epithelial cells: implications for immunotherapy of Clostridioides difficile infection
Source: Gut Microbes. 2021 Aug 25;13(1):1968258. doi: 10.1080/19490976.2021.1968258 (PMC8405154; doi:10.1080/19490976.2021.1968258)
Supplement: Supplemental Material [file KGMI_A_1968258_SM1813.zip › Supplementary information/Supplementary Table 1.docx]

**Supplementary Table 1.** Demographic and clinical characteristics of the study patients

|  | **Non-CDI patients**  **(n=63)** | **CDI patients**  **(n=119)** | ***p-*value** |
| --- | --- | --- | --- |
| **Gender** | | | |
| Male | 35 (55.6%) | 67 (56.3) | 0.638 |
| Age (year) | 59 ± 18 | 65 ± 16 | 0.373 |
| **Wards** | | | |
| Medical ward | 29 (46.0%) | 52 (43.7%) | 0.265 |
| Surgical ward | 23 (36.5%) | 40 (33.6%) |  |
| ICU | 11 (17.5%) | 27 (22.7%) |  |
| **Stool characteristics** |  |  |  |
| Watery | 32 (50.8%) | 71 (59.7%) | 0.212 |
| Mucoid | 12 (19.0%) | 24 (20.2%) | 0.811 |
| Bloody (gross) | 2 (3.2%) | 12 (10.1%) | 0.267 |
| All-cause mortality | 2 (3.2%) | 14 (11.8%) | 0.029 |
| **Laboratory indicators, median (IQR)** | | | |
| WBC [10^9^/l] | 8.6 (5.7-10.9) | 10.4 (8.1-19.0) | 0.003 |
| Neutrophil [10^9^/l] | 6.5 (4.9-12.1) | 8.9 (5.2-15.7) | 0.037 |
| Lymphocyte[10^9^/l] | 1.5 (1.0-2.2) | 1.2 (0.70-1.9) | 0.059 |
| Eosinophil [10^9^/l] | 0.1 (0.05-0.2) | 0.07 (0.01-0.1) | 0.031 |
| ALB [g/l] | 32.5 (29.8-35.3) | 29.7 (24.1-36.1) | 0.040 |
| CRP [mg/l] | 34.0 (16.2-78.2) | 40.3 (18.2-89.5) | 0.121 |
| PCT [ng/ml] | 0.2 (0.06-0.8) | 0.40 (0.1-2.2) | 0.022 |
| Cr [µmol/l] | 58.1 (47.8-71.5) | 71.6 (51.0-102.5) | 0.189 |
| ICU, Intensive Care Unit; IQ, interquartile; WBC, white blood cells; ALB, albumin; CRP, C-reactive protein; PCT, Procalcitonin; Cr, Creatinine; | | | |
|  |  |  |  |
