# Supplementary material for: IL-27 induces LL-37/CRAMP expression from intestinal epithelial cells: implications for immunotherapy of Clostridioides difficile infection
Source: Gut Microbes. 2021 Aug 25;13(1):1968258. doi: 10.1080/19490976.2021.1968258 (PMC8405154; doi:10.1080/19490976.2021.1968258)
Supplement: Supplemental Material [file KGMI_A_1968258_SM1813.zip › Supplementary information/Supplementary Table 3.docx]

**Supplementary Table 3. Correlation between fecal IL-27 and cytokine levels at the time of CDI diagnosis**

Variable IL-27

*r p-*value

IL-1β 0.103 0.110

TNF-α 0.019 0.404

IL-6 0.022 0.363

IL-8 0.011 0.567

LL-37 0.413 0.017
