# Supplementary figures and images for: IL-27 induces LL-37/CRAMP expression from intestinal epithelial cells: implications for immunotherapy of Clostridioides difficile infection
Source: Gut Microbes. 2021 Aug 25;13(1):1968258. doi: 10.1080/19490976.2021.1968258 (PMC8405154; doi:10.1080/19490976.2021.1968258)

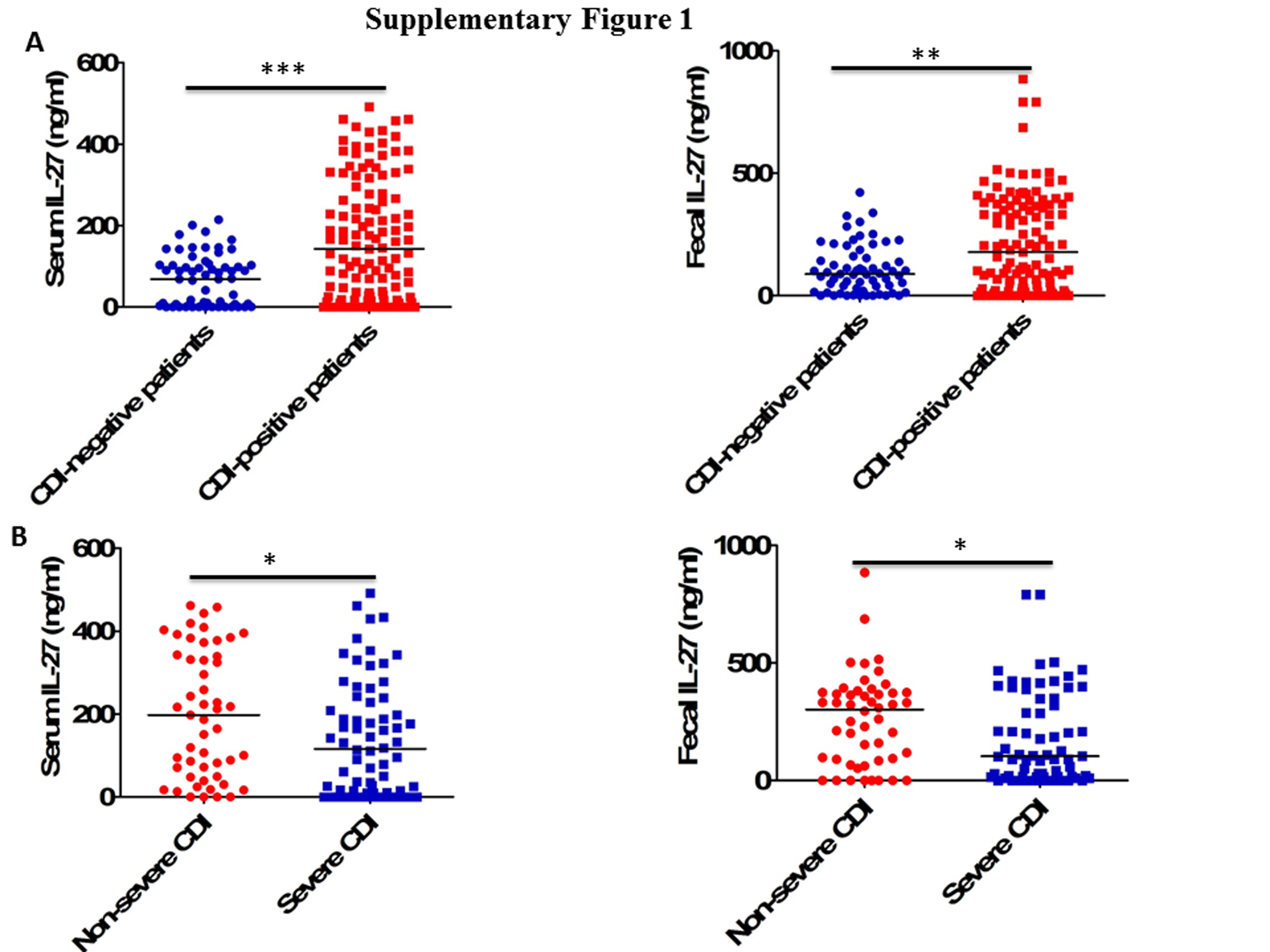

Supplement: Supplemental Material [file KGMI_A_1968258_SM1813.zip › Supplementary information/Supplementary Figure 1.TIF]
